# Supplementary material for: Post-covid medical complaints following infection with SARS-CoV-2 Omicron vs Delta variants
Source: Nat Commun. 2022 Nov 30;13:7363. doi: 10.1038/s41467-022-35240-2 (PMC9709355; doi:10.1038/s41467-022-35240-2)
Supplement: Supplementary file 1 — Supplementary information [file 41467_2022_35240_MOESM1_ESM.pdf]

Supplementary file for the paper
Post-covid medical complaints following infection with SARS-CoV-2 Omicron vs Delta variants

By Magnusson et al., 2022

Table of contents (brief titles):

Supplementary Table 1. Data sources in Beredt C19 used in this study and information obtained from each source. **p. 2**

Supplementary Figure 1. The day-by-day proportion (prop.) of screened tests that were confirmed as the SARS-CoV-2 Omicron or Delta variants, assessed for each of the 25 days included in the inclusion period. **p. 3**

Supplementary Table 2. Person-days, numbers of failures and incidence rates per 100 000 person-days by diagnosis in primary care from 14 to up to 126 days after test date for SARS-CoV-2. **p. 4**

Supplementary Table 3. Estimated hazard ratios of post-covid diagnoses in primary care from 14 to up to 126 days after testing for SARS-CoV-2, for persons with the omicron variant vs the delta variant. **p. 5**

Supplementary Table 4. Estimated hazard ratios of post-covid diagnoses in primary care from 14 to up to 126 days after testing for SARS-CoV-2, for persons with Omicron or Delta, both compared to persons testing negative. **p. 6**

Supplementary Figure 2. Risks of complaints from 14 to up to 126 days after SARS-CoV-2 infection with the omicron or delta variant, adjusted for age, sex, education, comorbidities, test and care activity and vaccination. **p. 7**

Supplementary Figure 3. Risks of complaints from 14 to up to 126 days after SARS-CoV-2 infection with the delta or omicron variant, adjusted for age, sex, education, comorbidities, test and care activity and vaccination, with censoring of observations from the date of positive test and onwards. **p. 8**

Supplementary Figure 4. Weekly proportions having post-covid complaints per 10 000 individuals, in the acute (14-29 days), sub-acute (30-89 days) and chronic (≥90 days) post-covid phases, as distinguished by vertical lines for days 14, 30 and 90, with censoring of observations from the date of positive test and onwards among non-infected. **p. 9**

Supplementary Figure 5. Weekly proportions having any post-covid complaint per 10 000 individuals, in the acute (14-29 days), sub-acute (30-89 days) and chronic (≥90 days) post-covid phases, as distinguished by vertical lines for days 14, 30 and 90, stratified by vaccination status. **p. 10**

Supplementary Table 5. The differences between the group being infected with Omicron and the group being infected with Delta in prevalence of different post-covid complaints over time, stratified by vaccination status. **p. 11**

Supplementary References **p. 12**

Supplementary Table 1. Data sources in Beredt C19 used in this study and information obtained from each source.

**Name of data source**	**Information obtained**
The National Population Register	Resident of Norway December 1st 2021<br>Age<br>Sex (assigned to all participants at birth and registered as male or female in the The National Population Register)<br>Birth country (Norway vs abroad)
Statistics Norway	Education status (no school, primary school, upper secondary school and college/university in four categories)
The Norwegian Surveillance System for Communicable Diseases	Date of sample of SARS-CoV-2 positive test<br>Classification of SARS-CoV-2 variants by targeted commercial or in-house PCR analyses for variant detection, Sanger sequencing of selected parts of the viral genome or whole genome sequencing.<br><br>For continuous surveillance purposes, 25% of SARS-CoV-2 positive samples or up to 100 samples per week per local laboratory is sent to a reference laboratory for whole genome sequencing. When Omicron emerged in Norway in late November 2021, the laboratories were requested to perform variant analyses locally on all positive samples. If this was not possible for capacity reasons, samples suspected to contain the Omicron variant was prioritized for variant analyses.
The National Immunization Register	Vaccination status with mRNA vaccines prior to test date, 2 or 3 doses.
Norway Control and Payment of Health Reimbursement (KUHR) Database	All cause-specific medical records (in-person and remote) registered in all Norwegian general practitioner and emergency wards, from 14 to up to 126 days after the PCR test date. The codes include diseases, disorders, signs, symptoms, and/or complaints as classified by the physician consulted.
The Norwegian Patient Register	The number of comorbidities, based on risk conditions for severe COVID-19 that were defined by an expert panel in ethics and prioritization for vaccination, with data identified in data from the Norwegian Patient Register (reference #1).<br>Hospitalization for severe COVID-19 from -2 to +14 days from the test date.

Information was linked at the individual level using the unique personal identification number (encrypted version) provided to every Norwegian resident at birth or upon immigration (reference #1).

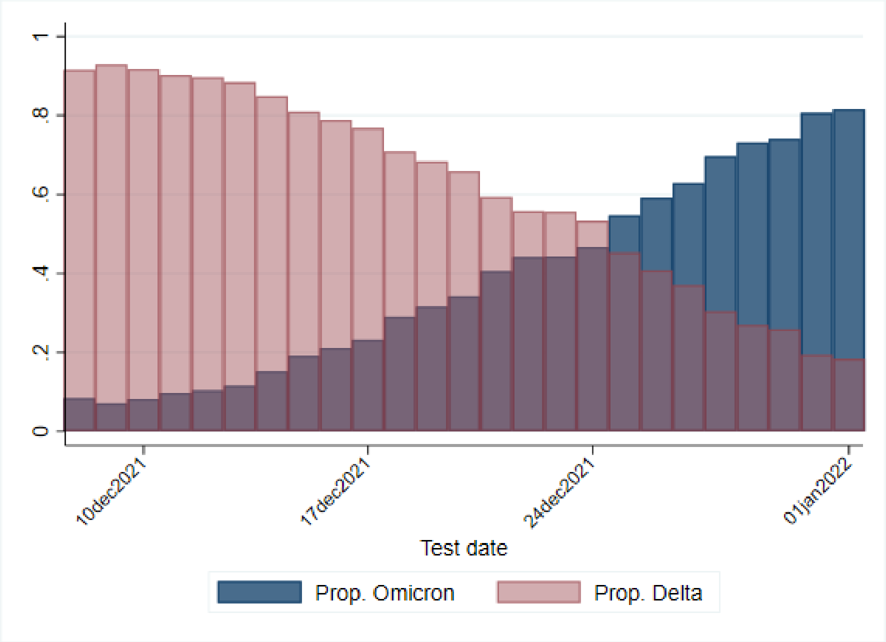

Supplementary Figure 1. The day-by-day proportion (prop.) of screened tests that were confirmed as the SARS-CoV-2 Omicron or Delta variants, assessed for each of the 25 days included in the inclusion period. Source data are provided as a Source Data file.

Supplementary Table 2. Person-days, numbers of failures and incidence rates per 100 000 person-days by diagnosis in primary care from 14 to up to 126 days after test date for SARS-CoV-2.

		SARS-CoV-2 omicron	SARS-CoV-2 delta	Tested negative without censoring*	Untested, without censoring*	Tested negative or untested, with censoring*
Any complaint	Person-days	1 349 804	2 360 779	10 660 007	122 000 000	124 800 000
	Failures	2240	4507	17 410	156 395	161 515
	Incidence rate	166	191	163	128	129
	(95%CI)	159-173	185-197	160-166	127-129	129-130
Musculoskeletal pain	Person-days	1 435 796	2 535 206	11 257 065	126 700 000	129 500 000
	Failures	1083	2246	9241	91 283	94 105
	Incidence rate	75	89	82	72	73
	(95%CI)	71-80	85-92	80-84	72-73	72-73
Fatigue	Person-days	1 459 971	2 595 895	11 600 767	130 900 000	133 500 000
	Failures	661	1254	3801	25 095	25 767
	Incidence rate	45	48	33	19	19
	(95%CI)	42-49	46-51	32-34	19-19	19-19
Cough	Person-days	1 488 372	2 643 932	11 714 347	131 700 000	134 300 000
	Failures	177	353	1543	9548	9 656
	Incidence rate	12	13	13	7	7
	(95%CI)	10-14	12-15	13-14	7-7	7-7
Heart palpitations	Person-days	1 492 734	2 653 465	11 757 502	139 000 000	134 400 00
	Failures	72	163	635	5528	5706
	Incidence rate	5	6	5	4	4
	(95%CI)	4-6	5-7	4-6	4-4	4-4
Shortness of breath	Person-days	1 491 170	2 647 117	11 759 958	131 900 000	134 400 000
	Failures	107	285	613	5137	5091
	Incidence rate	7	11	5	4	4
	(95%CI)	6-9	10-12	5-6	4-4	4-4
Anxiety/depression	Person-days	1 472 305	2 611 713	11 574 431	130 100 000	132 600 000
	Failures	423	884	3648	34772	36 362
	Incidence rate	29	34	32	27	27
	(95%CI)	26-32	32-36	30-33	26-27	27-27
Brain fog	Person-days	1 494 863	2 658 461	11 775 821	132 000 000	134 600 000
	Failures	29	49	231	1942	2023
	Incidence rate	2	2	2	1	2
	(95%CI)	1-3	1-2	2-2	1-2	1-2

Failures represent the first medical record registered at the general practitioner or emergency ward with the diagnoses in question (musculoskeletal pain, fatigue etc. assuming no competing risk between the different diagnoses), from 14 to up to 126 days after the test date.

Supplementary Table 3. Estimated hazard ratios of post-covid diagnoses in primary care from 14 to up to 126 days after testing for SARS-CoV-2, for persons with the omicron variant vs the delta variant.

Outcome	SARS-CoV-2 status	Crude	95% CI	Adjusted*	95% CI*	Adjusted* + adj. for vaccination	95% CI
Any complaint	Delta	1		1		1	
	Omicron	0.88	[0.83,0.93]	0.96	[0.91,1.01]	0.97	[0.92,1.02]
Musculoskeletal pain	Delta	1		1		1	
	Omicron	0.88	[0.81,0.95]	0.99	[0.92,1.07]	0.99	[0.91,1.06]
Fatigue	Delta	1		1		1	
	Omicron	1.02	[0.92,1.12]	1.02	[0.92,1.13]	1.04	[0.94,1.15]
Cough	Delta	1		1		1	
	Omicron	0.98	[0.81,1.18]	1.03	[0.85,1.24]	1.02	[0.84,1.24]
Heart palpitations	Delta	1		1		1	
	Omicron	0.85	[0.64,1.13]	0.84	[0.63,1.12]	0.83	[0.62,1.11]
Shortness of breath	Delta	1		1		1	
	Omicron	0.75	[0.60,0.95]	0.77	[0.61,0.97]	0.77	[0.60,0.97]
Anxiety/depression	Delta	1		1		1	
	Omicron	0.89	[0.79,1.01]	0.92	[0.81,1.04]	0.93	[0.83,1.05]
Brain fog	Delta	1		1		1	
	Omicron	1.09	[0.67,1.75]	1.1	[0.68,1.77]	1.07	[0.66,1.73]

*Adjusted for age, sex, education status, country of birth, the number of comorbidities, the number of negative tests prior to the selected test date and the number of primary care visits prior to the selected test date. Stratified on calendar week of testing.

Supplementary Table 4. Estimated hazard ratios of post-covid diagnoses in primary care from 14 to up to 126 days after testing for SARS-CoV-2, for persons with Omicron or Delta, both compared to persons testing negative.

Outcome	SARS-CoV-2 status	Crude	95% CI	Adjusted*	95% CI*	Adjusted* + adj. for vaccination	95% CI
Any complaint	Tested negative	1		1		1	
	Delta	1.16	[1.12,1.20]	1.08	[1.05,1.12]	1.07	[1.03,1.11]
	Omicron	1.02	[0.98,1.07]	1.04	[0.99,1.09]	1.03	[0.99,1.08]
Musculoskeletal pain	Tested negative	1		1		1	
	Delta	1.06	[1.01,1.11]	1.02	[0.97,1.07]	1.02	[0.97,1.07]
	Omicron	0.93	[0.87,0.99]	1.01	[0.94,1.08]	1	[0.94,1.07]
Fatigue	Tested negative	1		1		1	
	Delta	1.36	[1.28,1.45]	1.26	[1.18,1.34]	1.24	[1.16,1.33]
	Omicron	1.39	[1.27,1.51]	1.28	[1.18,1.40]	1.29	[1.18,1.40]
Cough	Tested negative	1		1		1	
	Delta	0.98	[0.87,1.10]	0.93	[0.82,1.04]	0.93	[0.83,1.05]
	Omicron	0.96	[0.81,1.12]	0.95	[0.81,1.12]	0.95	[0.81,1.12]
Heart palpitations	Tested negative	1		1		1	
	Delta	1.16	[0.98,1.38]	1.1	[0.92,1.31]	1.12	[0.93,1.34]
	Omicron	0.98	[0.76,1.27]	0.92	[0.72,1.19]	0.93	[0.72,1.20]
Shortness of breath	Tested negative	1		1		1	
	Delta	1.75	[1.52,2.02]	1.69	[1.46,1.96]	1.69	[1.46,1.96]
	Omicron	1.32	[1.07,1.64]	1.3	[1.04,1.61]	1.29	[1.04,1.61]
Anxiety/depression	Tested negative	1		1		1	
	Delta	1.06	[0.99,1.14]	1	[0.93,1.08]	0.98	[0.91,1.05]
	Omicron	0.95	[0.85,1.05]	0.92	[0.83,1.02]	0.91	[0.82,1.01]
Brain fog	Tested negative	1		1		1	
	Delta	1.03	[0.76,1.41]	0.89	[0.65,1.22]	0.92	[0.66,1.26]
	Omicron	1.12	[0.75,1.68]	0.98	[0.65,1.47]	0.98	[0.65,1.47]

*Adjusted for age, sex, education status, country of birth, the number of comorbidities, the number of negative test prior to the selected test date and the number of primary care visits prior to the selected test date. Stratified on calendar week of testing.

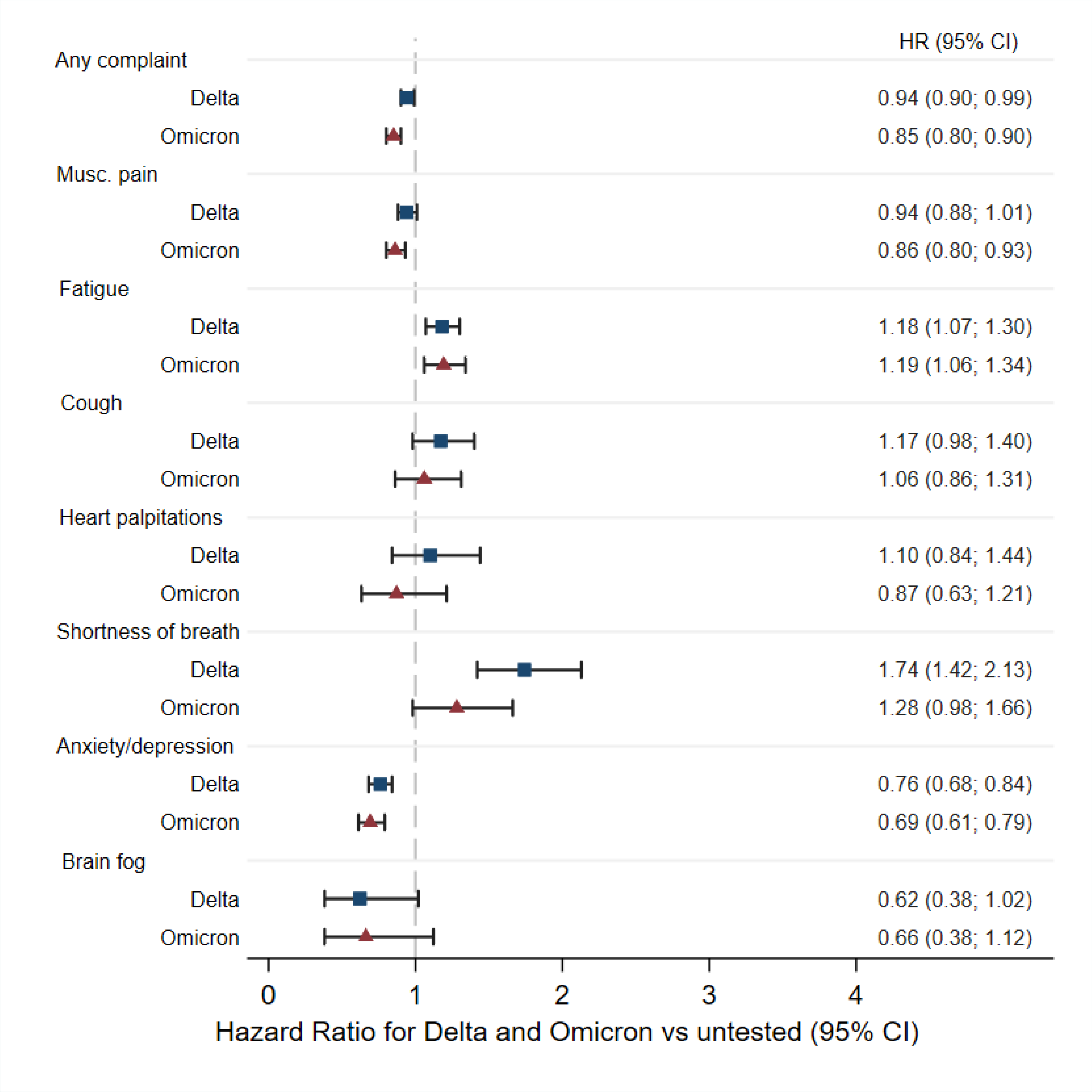

Supplementary Figure 2. Risks of complaints from 14 to up to 126 days after SARS-CoV-2 infection with the omicron or delta variant, adjusted for age, sex, education, comorbidities, test and care activity and vaccination. Reference category: persons who were never tested for SARS-CoV-2 prior to a randomly assigned test date (N=1 180 716) (dashed vertical line). Data are presented as Hazard Ratios (HR) with 95% confidence interval (CI) and examined over 8 independent experiments, one for each post-covid outcomes. Blue squares represent the estimates for the Delta variant compared to untested individuals. Red triangles represent the estimates for the Omicron variant compared to untested individuals. Musc. pain=musculoskeletal pain. Source data are provided as a Source Data file.

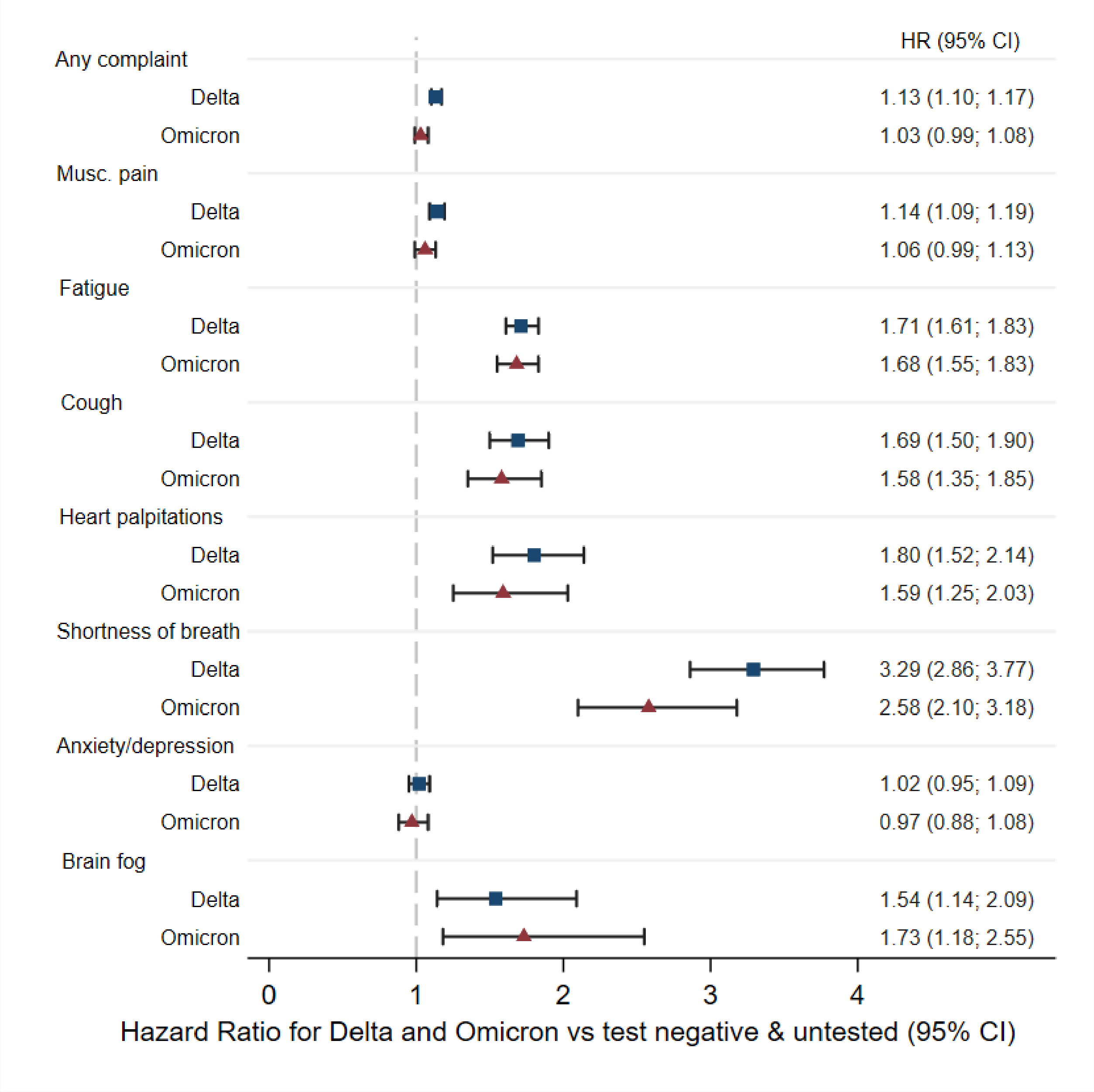

Supplementary Figure 3. Risks of complaints from 14 to up to 126 days after SARS-CoV-2 infection with the delta or omicron variant, adjusted for age, sex, education, comorbidities, test and care activity and vaccination, with censoring of observations from the date of positive test and onwards. Reference category: persons who tested negative in in the inclusion period and persons who were never tested for SARS-CoV-2 prior to a randomly assigned test date (N=1 286 013) (dashed vertical line). Blue squares represent the estimates for the Delta variant compared to individuals testing negative and untested individuals. Red triangles represent the estimates for the Omicron variant compared to individuals testing negative and untested individuals. Musc. pain=musculoskeletal pain. Data are presented as Hazard Ratios (HR) with 95% confidence interval (CI) and examined over 8 independent experiments, one for each post-covid outcomes. Source data are provided as a Source Data file.

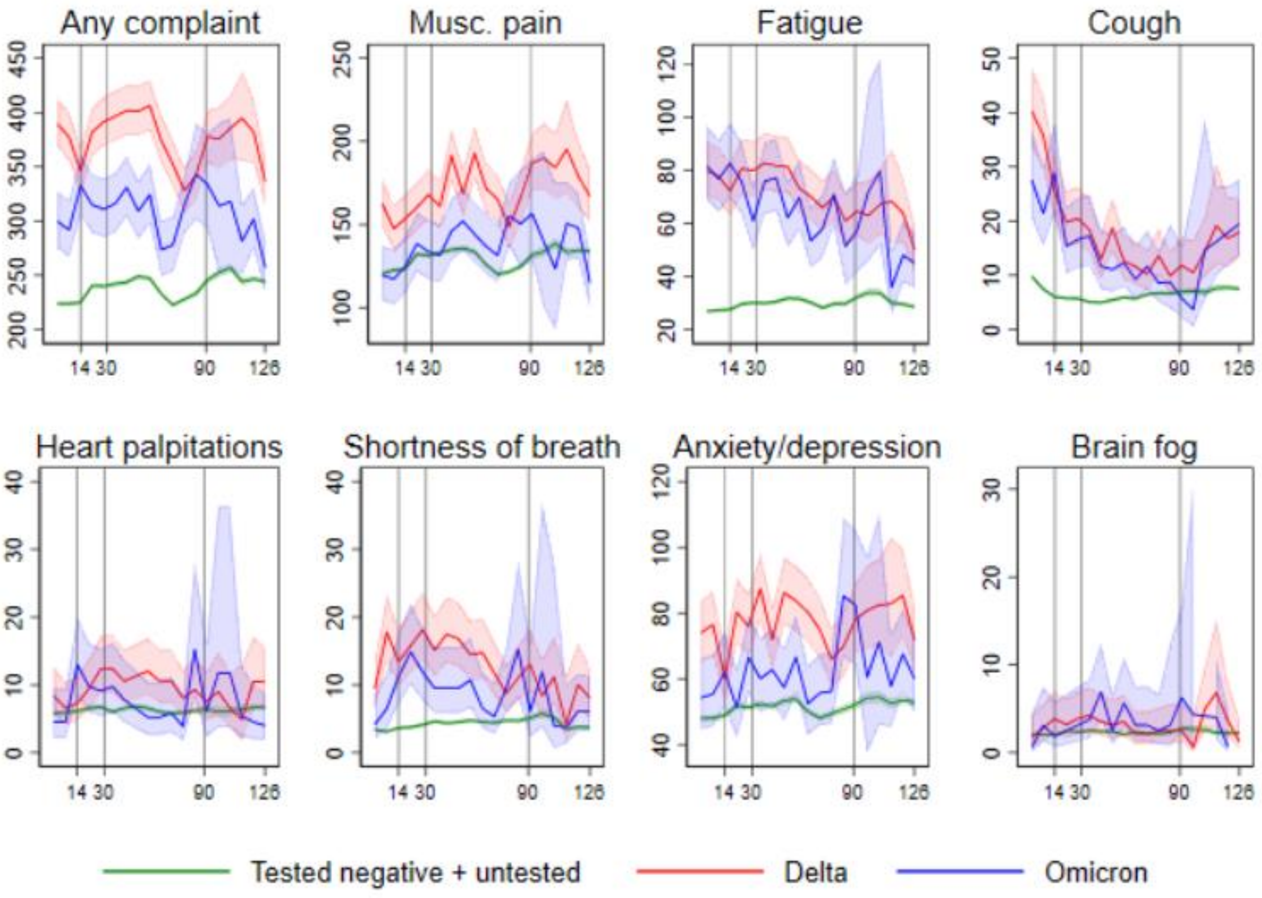

Supplementary Figure 4. Weekly proportions having post-covid complaints per 10 000 individuals, in the acute (14-29 days), sub-acute (30-89 days) and chronic (≥90 days) post-covid phases, as distinguished by vertical lines for days 14, 30 and 90, with censoring of observations from the date of positive test and onwards among non-infected. Data are presented as the number of individuals visiting primary care for the outcome in question at least once a week per 10 000 individuals in each group (colored lines), with 95% confidence intervals (shaded area). Estimates are predicted probabilities from a logit model with standard errors clustered on person level, adjusted for age, sex, education, comorbidities, test and care activity and vaccination. Source data are provided as a Source Data file.

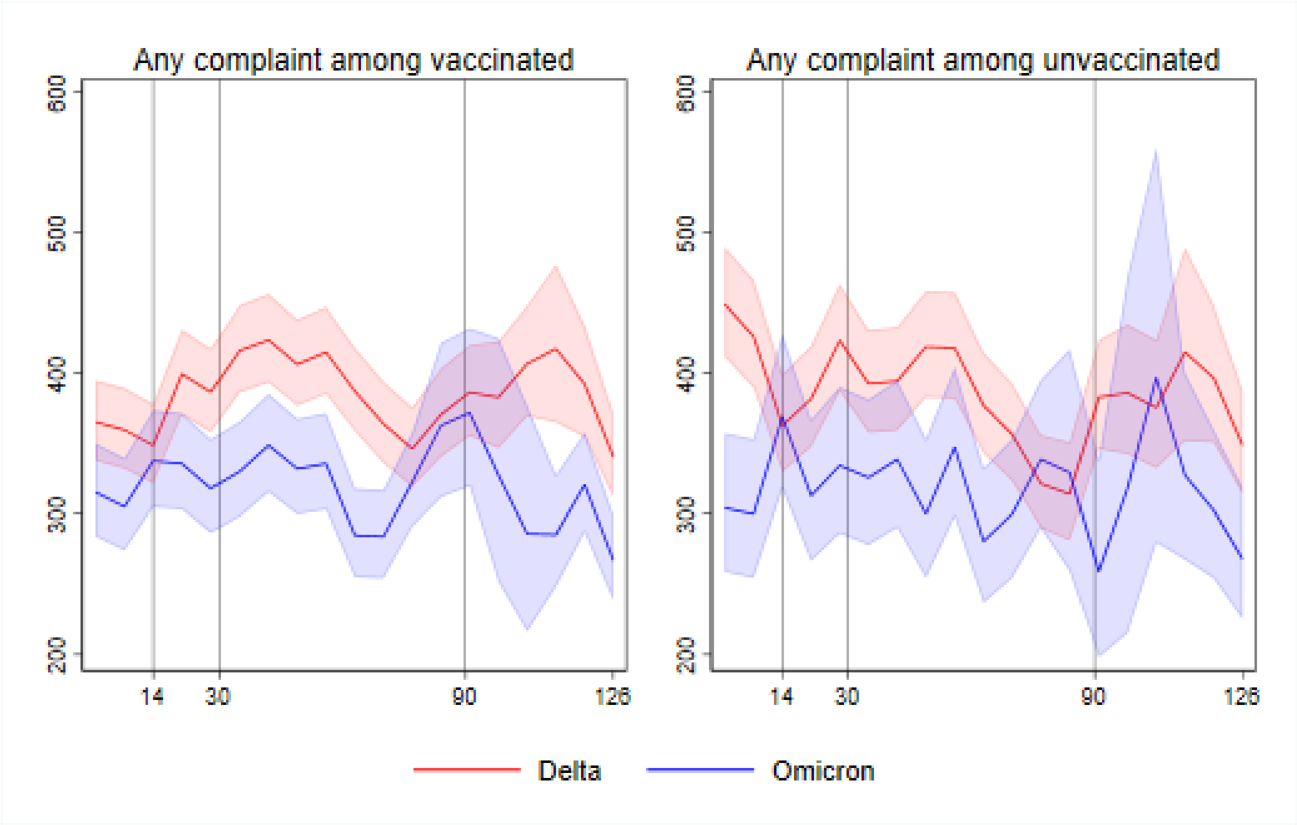

Supplementary Figure 5. Weekly proportions having any post-covid complaint per 10 000 individuals, in the acute (14-29 days), sub-acute (30-89 days) and chronic (≥90 days) post-covid phases, as distinguished by vertical lines for days 14, 30 and 90, stratified by vaccination status. Data are presented as the number of individuals visiting primary care for the outcome in question at least once a week per 10 000 individuals in each group (colored lines), with 95% confidence intervals (shaded area). The vaccinated individuals received the last dose 14-210 days prior to SARS-CoV-2 test (inclusion) date. The unvaccinated did not receive the last dose 14-210 days prior to SARS-CoV-2 test (inclusion) date. Estimates are predicted probabilities from a logit model with standard errors clustered on person level, adjusted for age, sex, education, comorbidities, test and care activity. Source data are provided as a Source Data file.

Supplementary Table 5. The differences between the group being infected with Omicron and the group being infected with Delta in prevalence of different post-covid complaints over time, stratified by vaccination status.

	Acute COVID-19	Acute post-covid period	Sub-acute post-covid period	Chronic post-covid period	Whole post-covid period
	**1-13 days**	**14-29 days**	**30-89 days**	**90 days and more**	**14 to 126 days**
Unvaccinated	-140	-43	-49	-81	-56
	-199 to -84	-97 to 11	-91 to -8	-129 to -33	-93 to -18
Vaccinated	-6	2	-22	-36	-20
	-47 to 35	-36 to 40	-53 to 8	-70 to -1	-5 to 7

Estimates are group differences in prevalence per 10 000 persons in the respective groups, with 95% confidence intervals, representing the group testing positive with Omicron minus the group testing positive with Delta, calculated from a logit model with standard errors clustered on person level, adjusted for age, sex, education, comorbidities, test and care activity. The vaccinated individuals received the last dose 14-210 days prior to SARS-CoV-2 test (inclusion) date. The unvaccinated did not receive the last dose 14-210 days prior to SARS-CoV-2 test (inclusion) date.

Supplementary References

1) Norwegian Institute of Public Health. The Norwegian Emergency Preparedness Register (BEREDT C19), 2020. https://www.fhi.no/en/id/infectious-diseases/coronavirus/emergency-preparedness-register-for-covid-19/
