# Supplementary material for: Post-covid medical complaints following infection with SARS-CoV-2 Omicron vs Delta variants
Source: Nat Commun. 2022 Nov 30;13:7363. doi: 10.1038/s41467-022-35240-2 (PMC9709355; doi:10.1038/s41467-022-35240-2)
Supplement: Supplementary file 2 — Description of Additional Supplementary files [file 41467_2022_35240_MOESM2_ESM.pdf]

Description of Additional Supplementary Files

File name: Supplementary Code 1

Description: STATA code for producing results in paper "Post-covid medical complaints following infection with SARS-CoV-2 Omicron vs Delta variants", by Magnusson et al., 2022.
