# Supplementary material for: Post-covid medical complaints following infection with SARS-CoV-2 Omicron vs Delta variants
Source: Nat Commun. 2022 Nov 30;13:7363. doi: 10.1038/s41467-022-35240-2 (PMC9709355; doi:10.1038/s41467-022-35240-2)
Supplement: Supplementary file 4 — Reporting Summary [file 41467_2022_35240_MOESM4_ESM.pdf]

nature portfolio

Corresponding author(s): Karin Magnusson

Last updated by author(s): Nov 2, 2022

Reporting Summary

Nature Portfolio wishes to improve the reproducibility of the work that we publish. This form provides structure for consistency and transparency in reporting. For further information on Nature Portfolio policies, see our Editorial Policies and the Editorial Policy Checklist.

Statistics

For all statistical analyses, confirm that the following items are present in the figure legend, table legend, main text, or Methods section.

n/a	Confirmed	
☐	☒	The exact sample size (n) for each experimental group/condition, given as a discrete number and unit of measurement
☐	☒	A statement on whether measurements were taken from distinct samples or whether the same sample was measured repeatedly
☐	☒	The statistical test(s) used AND whether they are one- or two-sided<br>*Only common tests should be described solely by name; describe more complex techniques in the Methods section.*
☐	☒	A description of all covariates tested
☐	☒	A description of any assumptions or corrections, such as tests of normality and adjustment for multiple comparisons
☐	☒	A full description of the statistical parameters including central tendency (e.g. means) or other basic estimates (e.g. regression coefficient) AND variation (e.g. standard deviation) or associated estimates of uncertainty (e.g. confidence intervals)
☒	☐	For null hypothesis testing, the test statistic (e.g. F, t, r) with confidence intervals, effect sizes, degrees of freedom and P value noted<br>*Give P values as exact values whenever suitable.*
☒	☐	For Bayesian analysis, information on the choice of priors and Markov chain Monte Carlo settings
☒	☐	For hierarchical and complex designs, identification of the appropriate level for tests and full reporting of outcomes
☐	☒	Estimates of effect sizes (e.g. Cohen's d, Pearson's r), indicating how they were calculated

Our web collection on statistics for biologists contains articles on many of the points above.

Software and code

Policy information about availability of computer code

Data collection	All computer codes to obtain the data relevant in this study were written and run in STATA MP v. 17. These data cannot be shared due to privacy restricitions, please see our data availability statement.
Data analysis	All computer codes used to analyse the data relevant in this study were written and run in STATA MP v. 17. The custom codes developed to reproduce the results are provided as an attachment to our paper.

For manuscripts utilizing custom algorithms or software that are central to the research but not yet described in published literature, software must be made available to editors and reviewers. We strongly encourage code deposition in a community repository (e.g. GitHub). See the Nature Portfolio guidelines for submitting code & software for further information.

Data

Policy information about availability of data

All manuscripts must include a data availability statement. This statement should provide the following information, where applicable:

- Accession codes, unique identifiers, or web links for publicly available datasets
- A description of any restrictions on data availability
- For clinical datasets or third party data, please ensure that the statement adheres to our policy

Source data are provided with this paper. The dataset of this study was the Emergency Preparedness Register for COVID-19 (Beredt C19), which is a property of the Norwegian Institute of Public Health that was provided to the researchers through a restricted-access agreement that prevents sharing the dataset with a third party or publicly. The raw Beredt C19 data are protected and are not available due to privacy law. Thus, individual-level data of patients included in this paper after

de-identification are considered sensitive and will not be shared. However, the individual-level data in the registries compiled in Beredt C19 are accessible to authorized researchers after ethical approval and application to "helsedata.no/en" administered by the Norwegian Directorate of eHealth. Data requests may be sent to "service@helsedata.no.

Human research participants

Policy information about studies involving human research participants and Sex and Gender in Research.

Reporting on sex and gender

We have not explicitly defined what is meant by sex or gender in our paper, however our study includes both men and women and it is clear throughout the paper that the findings apply to both men and women and all genders in between. Sex was assigned to all participants at birth and registered as male or female in the The National Population Register. This information is provided in the oveview of data sources in the supplementary file (S-Table 1). For a more inclusive terminology, we report our data as gender. Because of few outcome observations in some analyses and no indications that results would differ by sex or gender, no sex- or gender-stratified analyses were provided.

Population characteristics

Population characteristics are thorrougly described in our paper: Of in total 3 696 005 persons eligible for the study, 105 297 persons tested negative during our study period, and 57 727 persons had a positive test result that was screened for SARS-CoV-2 variant (Figure 1). Individuals infected with the Omicron variant (N=13 365) were generally younger, had higher education, fewer comorbidities and were more often vaccinated than individuals infected with the Delta variant (N=23 767) (Table 1). There were also some group differences in the amount of follow-up time by study group and by outcome, in the main analysis and in the sensitivity analysis (S-Table 2). Among individuals testing negative and individuals who were untested, 18 866 (17.9%) and 121 317 (10.3%) tested positive during follow-up and were non-censored in the main analyses (test negative) and in the sensitivity analyses (untested), respectively. When these two study groups were combined, 140 183 (10.9%) tested positive and were censored in analyses with censoring of observations from the date of positive test and onwards. The mortality during follow-up was low (0.07% (95% CI=0.03-0.13), 0.05% (95% CI=0.03-0.08, 0.09% (95% CI=0.08-0.12) and 0.14% (95%CI=0.14-0.15), for individuals infected with Omicron, Delta, individuals who tested negative or who were untested, respectively.

Recruitment

The recruitment process is thorrougly described in our paper: Using a prospective cohort study design applied to data in the Norwegian Emergency Preparedness Register (S-Table 1),19 we included all Norwegian residents aged 18-70 years, who tested negative or positive for SARS-CoV-2 with known variant during the period that the Omicron and Delta variants had the greatest overlap in Norway; from December 8th to December 31st 2021. These data were linked on the personal ID number to provide information on healthcare contacts in primary care (general practitioners and emergency wards) with specific medical record (S-Table 1). The PCR testing criteria were constant throughout the study period and included persons with symptoms of COVID-19, persons in close contact with anyone with COVID-19 as well as persons having a positive antigen test. Screening for SARS-CoV-2 variant was performed by Sanger or whole genome sequencing on all positive PCR tests if the laboratories had capacity and only on positive tests with suspected Omicron if the laboratory had capacity challenges. We excluded all individuals with previous positive PCR tests (up until December 7th 2021, to avoid pre-existing post-covid complaints), individuals with unscreened positive tests and all individuals who had a hospital contact from -2 to +14 days from the test date8. In this way, we could study individuals with known infection with SARS-CoV-2 Omicron and Delta variant with assumed mild disease courses and/or who were known not to be tested as part of hospital contact or routine testing at hospitals. Participants were categorized into three study groups based on their test result and date of testing: 1) individuals infected with Omicron, 2) individuals infected with Delta, and 3) individuals who were non-infected (tested negative during the study period and/or earlier but allowed to test positive after the test date).

As our study was based on register data, we had no self-selection bias. However, there were some important differences in baseline characteristics on seeking medical care (testing and health care use) and mortality that may impact on our findings through selection/collider stratification and/or confounder bias. We believe our methodological approach ensuring comparison of persons individuals who were tested in the same calendar week, the inclusion of untested and untested + test negative in sensitivity analyses, as well as the adjustment for a range of covariates including health-seeking behaviour would limit these potential biases.

Ethics oversight

Included in the paper: The Ethics Committee of South-East Norway confirmed (June 4th 2020, #153204) that external ethical board review was not required. The data sources (The emergency preparedness register for COVID-19 (Beredt C19)) and methods used were regarded by the ethical committee to respond to research aims not falling within the Law of Health Research §§ 2 and 4a. Their resolution was also based on the fact that the data sources were established and handled in accordance with the Health Preparedness Act §2-4 (11), enabling a quick and responsive way for the Norwegian government to access knowledge of how to handle the pandemic. No informed consent from participants was required since our study was based on routinely collected register data covering the entire Norwegian population. Data from the different registers included in the study were linked by the responsible researchers using an encrypted personal ID-variable. The researchers responsible for the data linkage and analyses had no access to the unencrypted ID-numbers. All methods were carried out in accordance with relevant guidelines and regulations. To protect participants privacy and security of personal data, all data were handled under strict confidentiality and access control as described in the Norwegian Institute of Public Health's internal documentation.

Also included briefly, in the Methods section: Institutional board review was conducted, and The Ethics Committee of South-East Norway confirmed (June 4th 2020, #153204) that external ethical board review was not required. No informed consent was required or obtained, and participants were not compensated for participation.

Note that full information on the approval of the study protocol must also be provided in the manuscript.

Field-specific reporting

Please select the one below that is the best fit for your research. If you are not sure, read the appropriate sections before making your selection.

- [x] Life sciences
- [ ] Behavioural & social sciences
- [ ] Ecological, evolutionary & environmental sciences

For a reference copy of the document with all sections, see nature.com/documents/nr-reporting-summary-flat.pdf

Life sciences study design

All studies must disclose on these points even when the disclosure is negative.

Sample size	We did not perform any sample size calculations and had no a priori requirement for the size of the sample. In observational studies performed posteriorly like ours, it is both recommended and general practice to report and focus on confidence intervals to provide information on statistical power and precision. The widths of confidence intervals imply sufficient statistical power and precision. However, estimates for brain fog had wide confidence interval. The inconclusiveness for this outcome is acknowledge in the Discussion section.
Data exclusions	Included in the manuscript: We excluded all individuals with previous positive PCR tests (up until December 7th 2021, to avoid pre-existing post-covid complaints), individuals with unscreened positive tests and all individuals who had a hospital contact from -2 to +14 days from the test date8. In this way, we could study individuals with known infection with SARS-CoV-2 Omicron and Delta variant with assumed mild disease courses and/or who were known not to be tested as part of hospital contact or routine testing at hospitals.
Replication	We had several sensitivity analyses that replicated our findings from the main analyses. Their methods are described and their results are presented in the results section and supplementary file.
Randomization	Randomization of individuals becomming infected with Omicron or Delta would not be acceptable from an ethical view and was not performed.
Blinding	Blinding was not relevant in our study as it was not an intervention study.

Reporting for specific materials, systems and methods

We require information from authors about some types of materials, experimental systems and methods used in many studies. Here, indicate whether each material, system or method listed is relevant to your study. If you are not sure if a list item applies to your research, read the appropriate section before selecting a response.

Materials & experimental systems

n/a	Involved in the study	
[x]	[ ]	Antibodies
[x]	[ ]	Eukaryotic cell lines
[x]	[ ]	Palaeontology and archaeology
[x]	[ ]	Animals and other organisms
[x]	[ ]	Clinical data
[x]	[ ]	Dual use research of concern

Methods

n/a	Involved in the study	
[x]	[ ]	ChIP-seq
[x]	[ ]	Flow cytometry
[x]	[ ]	MRI-based neuroimaging
